# DEEPrior: a deep learning tool for the prioritization of gene fusions

**DOI:** 10.1093/bioinformatics/btaa069

**Published:** 2020-02-04

**Authors:** Marta Lovino, Maria Serena Ciaburri, Gianvito Urgese, Santa Di Cataldo, Elisa Ficarra

**Affiliations:** b1 Department of Control and Computer Engineering; b2 Interuniversity Department of Regional and Urban Studies and Planning, Politecnico di Torino, Torino 10129, Italy

## Abstract

**Summary:**

In the last decade, increasing attention has been paid to the study of gene fusions. However, the problem of determining whether a gene fusion is a cancer driver or just a passenger mutation is still an open issue. Here we present DEEPrior, an inherently flexible deep learning tool with two modes (Inference and Retraining). Inference mode predicts the probability of a gene fusion being involved in an oncogenic process, by directly exploiting the amino acid sequence of the fused protein. Retraining mode allows to obtain a custom prediction model including new data provided by the user.

**Availability and implementation:**

Both DEEPrior and the protein fusions dataset are freely available from GitHub at (https://github.com/bioinformatics-polito/DEEPrior). The tool was designed to operate in Python 3.7, with minimal additional libraries.

**Supplementary information:**

Supplementary data are available at *Bioinformatics* online.

## 1 Introduction

Gene fusions are recently playing an important role in the study of cancer and some of them are even used as a diagnostic marker (Mertens *et al.*, 2015). The advent of next-generation sequencing technologies together with the increasing availability of fusion detection tools (Haas *et al.*, 2017; Iyer *et al.*, 2011; McPherson *et al.*, 2011) has allowed the identification of many candidate fusions both in oncological and healthy samples (Babiceanu *et al.*, 2016; Gao *et al.*, 2018). However, determining which gene fusions are drivers of cancer processes and not just passenger mutations is still an open and non-trivial problem. A first step toward the solution of this problem is taken by the fusion detection tools, that filter the candidate gene fusions based on the sample’s reads, trying to reduce as much as possible the number of false positives (i.e. detected gene fusions that are not found in later lab validation). A second step is taken by specific post-processing tools such as Oncofuse (Shugay *et al.*, 2013) and Pegasus (Abate *et al.*, 2014) to predict the oncogenic potential of a gene fusion. These tools are both based on traditional machine learning (ML) techniques trained with the protein domains of the fusion proteins. Although protein domains are very meaningful for the characterization of gene functions, the use of such information as a feature for the ML model requires to carefully process the protein domains from scratch every time the training database is updated with novel validated fusions. On top of that, in spite of a large number of gene fusion databases recently released, the lack of public benchmarks reporting proteins resulting from annotated and validated gene fusions is an additional issue.

To avoid labor-intensive processing of the protein domains during retraining, in our preliminary work (Lovino *et al.*, 2019) to perform gene fusion classification and prioritization we explored a deep-learning model directly trained with the amino acid sequences of the fusion proteins.

Here we present DEEPrior, a simple and easy-to-use tool for the prioritization of gene fusions based on a new and more sophisticated prediction model consisting of a Convolutional Neural Network (CNN) and a bidirectional Long Short Term Memory (LSTM) network to handle the prioritization problem. The deep-learning model is trained on the amino acid sequences of the fusion proteins.

In addition to the tool, we release to the scientific community a database with 4779 amino acid protein sequences that we collected and reconstructed for this work by combining the information reported by multiple sources (Babiceanu *et al.*, 2016; Forbes *et al.*, 2010; Lee *et al.*, 2017).

## 2 The tool

DEEPrior is a user-friendly tool for gene fusions prioritization downstream of gene fusion detection tools. It is implemented in Python 3.7 with minimal additional libraries, and it is available for both CPU and GPU. The tool supports two different modes.

1. Inference (default mode). This mode performs a prioritization of an input set of gene fusions, exploiting a given prediction model.

*Input:* an *N *×* *4 tabular file with rows corresponding to gene fusions each with four respective attributes: chromosome and coordinate of 5p′ end, chromosome and coordinate of 3p′ end. Alternatively, the user can provide as input directly the outcome of many gene fusion detection tools. The list of supported tools can be found in the Supplementary Material.

*Output:* a tabular file with *N* rows corresponding to gene fusions, where for each gene fusion an oncogenic probability value (a value in [0, 1] range) is provided, together with additional information, such as name, description and ENSEMBL identifier of 5p′ and 3p′ genes, and the following specific information about the fusion: length of the fused protein, whether the protein is predicted to be truncated, whether 3p or 5p gene is complete and the corresponding fusion protein sequence. The relevant fields of DEEPrior output can be found in Table 1 (see Supplementary Material).

**Table 1.** Relevant fields in the DEEPrior output file for the Inference mode

Fusion pair	Onc prob	5p gene info	3p gene info	Main protein length	Trunc protein	5p gene compl	3p gene compl	Main protein
TMPRSS2_ERG	0.80	…	…	123	Yes	No	Yes	PYMYSHEK..
RPS6KB1_SNF8	0.24	…	…	20	Yes	No	No	PGARRVRL…
ACACA_STAC2	0.88	…	…	70	Yes	No	No	WGIPLPW…

*Note*: Fusion pair indicates the common names of the genes involved in the fusion; Onc prob is the oncogenic probability value reported by the tool; Main protein length is the length of the fused protein; Trunc protein reports if the fused protein is truncated (an early stop codon occurs in the protein) or not; 5p gene comp indicates if 5p gene is complete in the fusion (stop codon of the upstream gene is present in the protein); 3p gene compl indicates if 3p gene is complete in the fusion (start codon of the downstream gene is present in the protein); Main protein is the protein reconstructed by DEEPrior. 5p and 3p gene info fields stand for a list of many other useful information about the genes involved in the fusion. The complete list is reported in the Supplementary Material.

2. Retraining. In case new validated gene fusions are available (e.g. new cancer or new gene fusion variants), this mode can be optionally employed to easily generate a custom prediction model.

*Input:* an *N *×* *5 tabular file, with rows corresponding to new gene fusions to be added to the prediction model. The required attributes are chromosome and coordinate of both 5p′ and 3p′ end, and a label of that gene fusion (0 for not oncogenic and 1 for oncogenic).

*Output:* a .*hdf5* file corresponding to the newly generated model. The new model can further be selected as model in the Inference mode.

## 3 Approach

The workflow implemented by the inference mode is illustrated in Supplementary Figure S1.

After executing a fusion detection tool, for each gene fusion DEEPrior constructs all possible proteins (all coding transcripts of each gene are considered). All resulting amino acid sequences are then fed into the prediction model, which provides a score for each sequence. The final oncogenic probability value of the gene fusion is obtained as the maximum among these scores. By doing so, even a single protein with a score is sufficient to give a high oncogenic value to the gene fusion. The closer to 1, the higher the probability of the gene fusion being oncogenic. In the end, gene fusions are sorted based on the oncogenic probability value. According to his needs, the user can set a threshold *thr* in the range [0, 1] so that only fusions with a value ≥*thr* are considered as relevant. The prediction model consists of a CNN followed by a bidirectional LSTM network. The default inference model was trained on a training set made up of 2118 validated sequences, equally balanced between oncogenic and not oncogenic (see Data and Model parameters sections in the Supplementary Material).

## 4 Performance

The following refers to the GPU version, however similar results can be obtained with the CPU version, as they share the same architecture. The experiments were performed on two different datasets, namely Datasets 1 and 2. To assess the performance of DEEPrior, we first exploited Dataset 1, which is completely independent from the training set. It consists of 156 fusions, 122 oncogenic and 34 not oncogenic (see Supplementary Material). To decide whether a gene fusion is relevant, we set a threshold *thr* = 0.8 on the oncogenic probability value returned by the tool. By doing so, we obtained that 39.74% of the predictions were selected as relevant. Among them, 9.67% were false positives. To assess the goodness of this result, we run on the same dataset Oncofuse and Pegasus which provide a score of relevance in the range [0, 1]. To be consistent with our test, we set *thr* = 0.8. Oncofuse returned 10.71% of the fusions with 6.67% of false positives. Pegasus returned 8.97% of gene fusions, with 0 false positives.

In addition, we evaluated DEEPrior on Dataset 2, consisting of 2623 oncogenic gene fusions from the TCGA validated via WGS (see Supplementary Material). DEEPrior provided 32.48% of the fusions above the threshold, against the 23.55% of Oncofuse and the 15.36% of Pegasus.

## 5 Case study

To assess the relevance of our results, we first applied DEEPrior to 6 RNA-seq samples of breast cancer published by Edgren *et al.* (2011). We processed the samples using STAR-fusion (Haas *et al.*, 2017) and then DEEPrior with *thr* = 0.8. DEEPrior identified nine gene fusions as highly probable oncogenic. Six of them were reported in the original study (Edgren *et al.*, 2011) as validated. Concerning the remaining three gene fusions, we have to remark that any experiment for their validation was provided in the original study. In addition, we evaluated DEEPrior performance onto 4 RNA-seq samples of prostate cancer studied by Wu *et al.* (2012). In this case, DEEPrior identified TMPRSS2_ERG gene fusion as highly probable oncogenic. This fusion was validated by Wu *et al.* (2012) and its functional impact in prostate cancer is well known.

More information about sample accessions and validated gene fusions can be found in Supplementary Material.

## 6 Conclusions

DEEPrior is able to prioritize gene fusions from different tumors, by only exploiting the amino acids sequence of the fused proteins. Unlike the state-of-the-art tools, it also supports easy retraining and readaptation of the model.

## Supplementary Material

btaa069_Supplementary_Data
