# Supplementary material for: DEEPrior: a deep learning tool for the prioritization of gene fusions
Source: Bioinformatics. 2020 Feb 4;36(10):3248–50. doi: 10.1093/bioinformatics/btaa069 (PMC7214024; doi:10.1093/bioinformatics/btaa069)
Supplement: btaa069_Supplementary_Data [file btaa069_supplementary_data.pdf]

Supplementary Material
DEEPrior: a deep learning tool for the prioritization of gene fusions

M. Lovino*, M. Ciaburri*, G. Urgese+, S. Di Cataldo*, E. Ficarra*

*Department of Control and Computer Engineering, Politecnico di Torino, 10129, Italy
+Interuniversity Department of Regional and Urban Studies and Planning, Politecnico di Torino, 10129, Italy

1 DEEPrior input

1.1 Inference mode

DEEPrior is designed to support as input to the *Inference mode* the most popular gene fusion detection tools.
Supported tools are:

- ChimPIPE [1]
- DeFuse [2]
- EricScript [3]
- FusionCatcher [4]
- InFusion [5]
- JAFFA [6]
- SOAPfuse [7]
- STAR-Fusion [8]
- TopHat [9]

The user can therefore choose among the most popular gene fusion detection tools with no effort at all. However, we underline that any gene fusion of which the genomic breakpoints are known can be processed providing in a tab-separated file the genomic coordinates of the breakpoints. The first two columns refer to chromosome number and breakpoint coordinate of 5p gene, while third and fourth columns refer to 3p gene. Coordinates can be entered in genome version grch37 or grch38. An example of the general input file format is reported in Table 1.

chr5p	coord5p	chr3p	coord3p
chr7	1000000	chr4	1000000
chr9	2555965	chr6	56444888

Table 1. Example of the general input file format, in case the user would like to process gene fusions obtained with a gene fusion detection tool different than the supported ones. The first two columns refer to chromosome number and breakpoint coordinate of 5p gene, while third and fourth columns refer to 3p gene.

1.2 Retraining mode

In this case, the input file is a tab separated file and contains validated gene fusions to be included in the retraining of the model for which the label (oncogenic or not oncogenic) is known. The file is similar to the one reported in Table 1 and in addition it contains the *Label* column which indicates the class to which that gene fusion belongs. 0 means not oncogenic and 1 oncogenic. An example of this file is provided in Table 2.

chr5p	coord5p	chr3p	coord3p	label
chr7	1000000	chr4	1000000	0
chr9	2555965	chr6	56444888	1

Table 2. Example of the input file in the retraining mode, in case the user would like to include in the prediction model new validated gene fusions (e.g. a new cancer or new gene fusion variants) The first two columns refer to chromosome number and breakpoint coordinate of 5p gene, while third and fourth columns refer to 3p gene. *label* column must be 0 if the gene fusion is related to the not oncogenic class, 1 otherwise.

2 DEEPrior output

2.1 Inference mode

The output file contains the following information:

- **fusion_pair**: name of the gene fusion with common gene names
- **oncogenic probability value**: oncogenic probability value reported by the tool. It is a number between 0 and 1. Closer is the number to 1, higher is the probability to be oncogenic
- **version**: grch37 or grch38 depending on the genome version parameter defined during the running of DEEPrior. Remember that hg19 is equivalent to grch37 and hg38 is equivalent to grch38
- **chr5p**: chromosome number of 5p gene
- **coord5p**: breakpoint coordinate of 5p gene on chromosome 5p (1-based coordinate system)

- **5p strand**: strand of 5p gene
- **5p common name**: common name of 5p gene
- **5p ensg**: ENSEMBL gene identifier of 5p gene
- **5p gene functionality**: functionality of 5p gene (e.g. proteing coding or not)
- **5p gene description**: additional information about 5p gene provided by ENSEMBL, usually a description of the biological process in which the gene is involved
- **chr3p**: chromosome number of 3p gene
- coord3p: breakpoint coordinate of 3p gene on chromosome 3p (1-based coordinate system)
- **3p strand**: strand of 3p gene
- **3p common name**: common name of 3p gene
- **3p ensg**: ENSEMBL gene identifier of 3p gene
- **3p gene functionality**: functionality of 3p gene (e.g. protein coding or not)
- **3p gene description**: additional information about 3p gene provided by ENSEMBL, usually a description of the biological process in which the gene is involved
- **MainProteinLength**: length of the fused protein
- **TruncatedProtein**: Yes if the fused protein is truncated (an early stop codon occurs in the protein). No otherwise.
- **5p_gene_complete**: Yes if 5p gene is complete in the fusion (stop codon in upstream gene is present in the protein). No otherwise.
- **3p_gene_complete**: Yes if 3p gene is complete in the fusion (start codon in downstream gene is present in the protein). No otherwise.
- **main protein**: the protein with no skipped exons

2.2 Retraining mode

The retraining mode output consists of a *.hdf5* file containing the weights and the architecture of the new trained model. This model can then be used to perform the gene fusions inference instead of the default deep learning model.

3 Data

Although recently a large amount of databases related to gene fusions have been released, the availability of databases reporting the proteins resulting from annotated and validated gene fusions is still a critical issue. Here (https://github.com/bioinformatics-polito/DEEPrior/tree/master/DEEPrior/data), we release the protein fusions data-sets specifically reconstructed from multiple sources and used to asses DEEPrior performances to the community. Overall, we used three data-sets (one for training and two different ones for performance assessment), described with more details in the following. A label is associated to each gene fusion of the data-sets, respectively *Onco* for the oncogenic and *NotOnco* for the not oncogenic. We defined a fusion pair as the union of the 5p gene name with the 3p one.

3.1 Training set:

This set consists of 786 fusion pairs and 2118 sequences, respectively 1059 *Onco* and 1059 *NotOnco*, obtained from two different sources. The *Onco* sequences were obtained from COSMIC, Catalog of Somatic Mutations in Cancer [10]. Among all the mutations involved in oncogenic processes, COSMIC also provides a list of validated gene fusions in the Complete Fusion Export Table. Among all the instances reported, we selected only the ones for which complete information was provided about the transcripts and the exact breakpoint positions, in order to be able to reconstruct the resulting amino-acid sequence. The *NotOnco* sequences, on the other hand, were obtained from a work by Babicenau et al.[11], where more than 10000 gene fusions were obtained by applying SOAPfuse gene fusions detection tool to 171 non-neoplastic tissues. Among all the gene fusions reported in the paper, we first discarded the ones not belonging to the human species or coming from cell lines (ESC, MSC, MFC10). As the *NotOnco* gene fusions were over-represented respect to the *Onco* ones by one order of magnitude, we selected the *NotOnco* gene fusions that were present in at least four different tissues or different patients. To complete the dataset we added gene fusions that were present in at least three different tissues or different patients. The selection proceeded recursively until we obtained a total number of *NotOnco* sequences equal to the number of the *Onco* sequences.

3.2 Data-set 1:

This set ws used to test DEEPrior performances and it is composed of a total of 142 fusion pairs and 156 gene fusions,122 *Onco* and 34 *NotOnco*. As there are no fusion pairs in common with the training set, this set is completely statistically independent.

Overall, the data were extracted from three different sources.

The sequences associated with *Onco* gene fusions were extracted from the ChimerDB2.0 database [12]. The genomic positions were obtained by taking the gene fusions from ChimerDB3.0-ChimerSeq [13] that originate from ChimerDB2.0. Oncofuse and Pegasus were originally trained also on ChimerDB2.0 database.

33 of the *NotOnco* gene fusions were the false positives reported by TopHat-Fusion [9]. They were obtained from two healthy samples (testis and thyroid), with corresponding data published by Illumina within the BodyMap 2.0 project. The other *NotOnco* gene fusions were obtained by applying STAR-

Fusion on the Illumina BodyMap 2.0 [14] samples for which information about the originating tissue was provided.

3.3 Data-set 2:

This set was used to test DEEPrior performances and it is composed of 2595 fusion pairs and 2623 gene fusions, all belonging to the *Onco* category. This dataset was built starting from the work of Gao et al.[15], who published a fusion call set of more than 25000 gene fusions, obtained by applying three fusion detection tools on the entire TCGA database and appropriately filtering the fusions that are found in healthy samples. In addition, for the samples for which WGS data were available, the presence of gene fusions was validated at the DNA level. The validated gene fusions dataset was kindly provided by the Authors on request. The 1,78% of fusion pairs are in common with the training set.

4 DEEPrior workflow

DEEPrior workflow is summarized in the Figure 1.

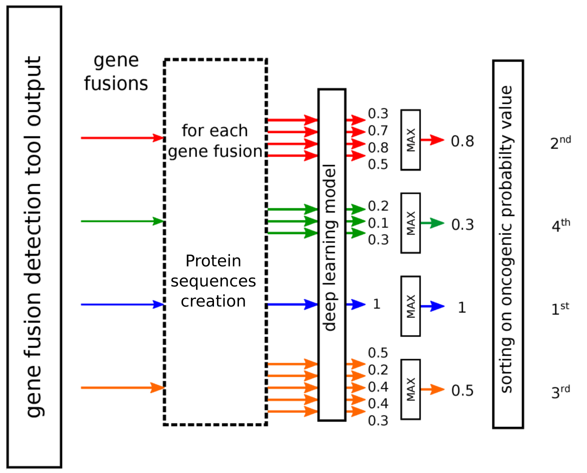

Figure 1. **Workflow of DEEPrior tool.** For each gene fusion (see different colors in the figure), DEEPprior generates all possible proteins, considering all transcripts of the fused genes. In the end, the amino-acid sequences are fed into the deep learning model, obtaining a 0-1 value for each protein. The oncogenic probability of each gene fusion is obtained as the maximum of all these values.

After executing a fusion detection tool, for each gene fusion DEEPrior constructs all possible proteins (all coding transcripts of each gene are considered). All resulting amino-acid sequences are then fed into the prediction model, which provides a score for each sequence. The final oncogenic probability value of the gene fusion is obtained as the maximum among these scores.

5 Model parameters

The model consists of a CNN followed by a bidirectional LSTM, trained on the entire training set. Data representation leverages on top of a token embedding learnt during the training, where the tokens (i.e. the individual amino acids) are mapped onto a geometric space so that similar tokens are geometrically close.

The model processes sequences between 6 and 4000 amino acids in length. Shorter sequences are not considered as they can hardly be functional, while sequences longer than 4000 amino acids are truncated before being processed by the model, since only the 0,22% of Uniprot sequences are longer than 4000 aminoacids. Furthermore, as the model has been defined, all sequences undergo a padding process.

To optimize the model, different configurations of number of layers, nodes per layer and dropout were evaluated, running 10-fold crossvalidation for each configuration and repeating each fold 10 times in order to establish the dependence on the initialization. In the end, the optimal model was the following. Embedding layer initialized randomly normal with size 16; One-dimensional convolution layer with 128 filters with size 5 kernel and Relu activation function. Max pooling with 3 window size and 0.3 dropout. Bidirectional LSTM with 32 nodes with tanh activation function and 0.3 dropout. Final dense layer with sigmoid activation function.

Number of epochs was set to 100, batch size to 64. In the training phase we used Keras callback EarlyStopping with patience (number of epochs with no improvement after which training will be stopped) equal to 30 and minimum change in the monitored quantity to qualify as an improvement equal to 0.

The network was implemented in Python 3.7 with Keras library [16] and its architecture is summarized in Figure 2.

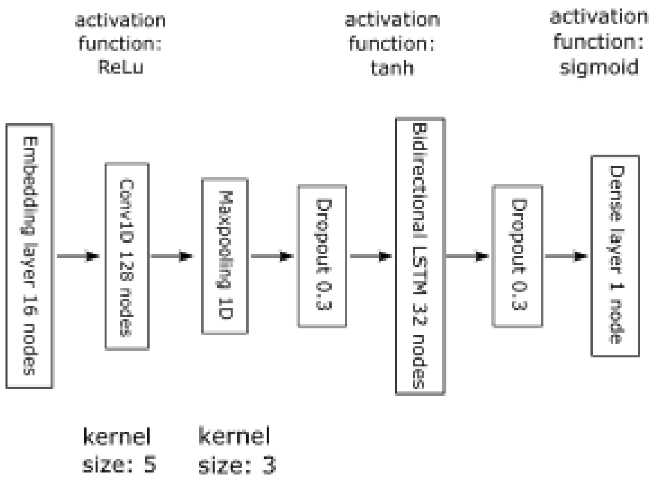

Figure 2. Architecture of the deep learning model in DEEPrior.

6 Additional experiments

In this section we report the details of the additional experiments of both the case studies and an additional set of *NotOnco* gene fusions.

6.1 Case study

We selected two well known studies to asses DEEPrior performances: 6 breast cancer samples [17] and 4 prostate cancer samples [18]. The samples are all RNA-seq data and are processed with STAR-fusion and then with DEEPrior. The SRA accession number of each sample as well as highly probable oncogenic gene fusions identified by DEEPrior ($thr = 0.8$) are reported in Table 3. Note that *Unknown* label in the *Validated* column means that the gene fusion was not considered for validation in studies [17] and [18].

tissue	SRA	Gene Fusion	Validated
breast	SRR064286	BCAS4_BCAS3	Yes
breast	SRR064287	BSG_NFIX	Yes
breast	SRR064287	PPP1R12A_SEPTIN10	Yes
breast	SRR064438<br>SRR064439	ACACA_STAC2	Yes
breast	SRR064438	LAMP1_MCF2L	Yes
breast	SRR064438<br>SRR064439	PIP4K2B_RAD51C	Unknown
breast	SRR064440<br>SRR064441	TATDN1_GSDMB	Unknown
breast	SRR064440<br>SRR064441	CYTH1_EIF3H	Yes
breast	SRR064440	ATAD5_TLK2	Unknown
prostate	SRR496597		
prostate	SRR496595		
prostate	SRR496581<br>SRR496580	TMPRSS2_ERG	Yes

Table 3. Sample tissue type (breast or prostate), sample SRA accession, highly probable oncogenic gene fusion identified by DEEPrior in that sample and validated label. More in detail, we checked if the reported gene fusion has been validated in studies [17] and [18]. *Unknown* label in the *Validated* column means that the gene fusion was not considered for validation in studies [17] and [18].

For breast cancer tissue, 9 gene fusions were identified as highly probable oncogenic and 6 of them are reported in the original study [17]. as validated. We have to remark that concerning the remaining 3 gene fusions the validation information was not available in [17]. On the other hand, on prostate cancer samples, DEEPrior identified TMPRSS2_ERG gene fusion as highly probable oncogenic. This fusion was validated by [18] and its functional impact in prostate cancer is well known.

6.2 *NotOnco* dataset

Since in the real world the number of not oncogenic gene fusions is at least one order of magnitude greater than the number of oncogenic gene fusions, we additionally tested the performance of DEEPrior on a set of not oncogenic gene fusions published by Babicenau et al. [11]. We selected a total of 5436 not oncogenic gene fusions. These fusions were not included in the training set, and occurred only once among all samples and all tissues. DEEPrior identified as not oncogenic the 75,02% of the gene fusions. Almost 80% of these fusions were predicted to be strongly not oncogenic (oncogenic probability value $<= 0.2$). These results suggested that DEEPrior is able to filter out the largest portion of the not oncogenic fusions.

Acknowledgements

We thank Gao et al.[15] for providing WGS validated data and Wen-Wei Liang for illustrating the WGS validation process.

Funding

This research received no external funding.

References

[1] Bernardo Rodríguez-Martín, Emilio Palumbo, Santiago Marco-Sola, Thasso Griebel, Paolo Ribeca, Graciela Alonso, Alberto Rastrojo, Begoña Aguado, Roderic Guigó, and Sarah Djebali. Chimpipe: Accurate detection of fusion genes and transcription-induced chimeras from rna-seq data. *BMC genomics*, 18(1):7, 2017.

[2] Andrew McPherson, Fereydoun Hormozdiari, Abdalnasser Zayed, Ryan Giuliany, Gavin Ha, Mark GF Sun, Malachi Griffith, Alireza Heravi Moussavi, Janine Senz, Nataliya Melnyk, et al. defuse: an algorithm for gene fusion discovery in tumor rna-seq data. *PLoS computational biology*, 7(5):e1001138, 2011.

[3] Matteo Benelli, Chiara Pescucci, Giuseppina Marseglia, Marco Severgnini, Francesca Torricelli, and Alberto Magi. Discovering chimeric transcripts in paired-end rna-seq data by using ericscript. *Bioinformatics*, 28(24):3232–3239, 2012.

[4] Daniel Nicorici, Mihaela Satalan, Henrik Edgren, Sara Kangaspeska, Astrid Murumagi, Olli Kallioniemi, Sami Virtanen, and Olavi Kilkku. Fusioncatcher-a tool for finding somatic fusion genes in paired-end rna-sequencing data. *BioRxiv*, page 011650, 2014.

[5] Konstantin Okonechnikov, Aki Imai-Matsushima, Lukas Paul, Alexander Seitz, Thomas F Meyer, and Fernando Garcia-Alcalde. Infusion: advancing discovery of fusion genes and chimeric transcripts from deep rna-sequencing data. *PloS one*, 11(12):e0167417, 2016.

[6] Nadia M Davidson, Ian J Majewski, and Alicia Oshlack. Jaffa: High sensitivity transcriptome-focused fusion gene detection. *Genome medicine*, 7(1):43, 2015.

[7] Wenlong Jia, Kunlong Qiu, Minghui He, Pengfei Song, Quan Zhou, Feng Zhou, Yuan Yu, Dandan Zhu, Michael L Nickerson, Shengqing Wan, et al. Soapfuse: an algorithm for identifying fusion transcripts from paired-end rna-seq data. *Genome biology*, 14(2):R12, 2013.

[8] Brian Haas, Alexander Dobin, Nicolas Stransky, Bo Li, Xiao Yang, Timothy Tickle, Asma Bankapur, Carrie Ganote, Thomas Doak, Natalie Pochet, et al. Star-fusion: fast and accurate fusion transcript detection from rna-seq. *BioRxiv*, page 120295, 2017.

[9] Daehwan Kim and Steven L Salzberg. Tophat-fusion: an algorithm for discovery of novel fusion transcripts. *Genome biology*, 12(8):R72, 2011.

[10] Simon A Forbes, Nidhi Bindal, Sally Bamford, Charlotte Cole, Chai Yin Kok, David Beare, Mingming Jia, Rebecca Shepherd, Kenric Leung, Andrew Menzies, et al. Cosmic: mining complete cancer genomes in the catalogue of somatic mutations in cancer. *Nucleic acids research*, 39(suppl_1):D945–D950, 2010.

[11] Mihaela Babiceanu, Fujun Qin, Zhongqiu Xie, Yuemeng Jia, Kevin Lopez, Nick Janus, Loryn Facemire, Shailesh Kumar, Yuwei Pang, Yanjun Qi, et al. Recurrent chimeric fusion rnas in non-cancer tissues and cells. *Nucleic acids research*, 44(6):2859–2872, 2016.

[12] Pora Kim, Suhyeon Yoon, Namshin Kim, Sanghyun Lee, Minjeong Ko, Haeseung Lee, Hyunjung Kang, Jaesang Kim, and Sanghyuk Lee. Chimerdb 2.0a knowledgebase for fusion genes updated. *Nucleic acids research*, 38(suppl_1):D81–D85, 2009.

[13] Myunggyo Lee, Kyubum Lee, Namhee Yu, Insu Jang, Ikjung Choi, Pora Kim, Ye Eun Jang, Byounggun Kim, Sunkyu Kim, Byungwook Lee, et al. Chimerdb 3.0: an enhanced database for fusion genes from cancer transcriptome and literature data mining. *Nucleic acids research*, 45(D1):D784–D789, 2016.

[14] Ensembl. The illumina body map 2.0 project. `https://www.ebi.ac.uk/arrayexpress/experiments/E-MTAB-513/`, 2010. Accessed: 2019-06-20.

[15] Qingsong Gao, Wen-Wei Liang, Steven M Foltz, Gnanavel Mutharasu, Reyka G Jayasinghe, Song Cao, Wen-Wei Liao, Sheila M Reynolds, Matthew A Wyczalkowski, Lijun Yao, et al. Driver fusions and their implications in the development and treatment of human cancers. *Cell reports*, 23(1):227–238, 2018.

[16] François Chollet et al. Keras, 2015.

[17] Henrik Edgren, Astrid Murumagi, Sara Kangaspeska, Daniel Nicorici, Vesa Hongisto, Kristine Kleivi, Inga H Rye, Sandra Nyberg, Maija Wolf, Anne-Lise Borresen-Dale, et al. Identification of fusion genes in breast cancer by paired-end rna-sequencing. *Genome biology*, 12(1):R6, 2011.

[18] Chunxiao Wu, Alexander W Wyatt, Andrew McPherson, Dong Lin, Brian J McConeghy, Fan Mo, Robert Shukin, Anna V Lapuk, Steven J M. Jones, Yongjun Zhao, et al. Poly-gene fusion transcripts and chromothripsis in prostate cancer. *Genes, Chromosomes and Cancer*, 51(12):1144–1153, 2012.
